# Clinical features and ^18^F-FDG PET/CT for distinguishing of malignant lymphoma from inflammatory lymphadenopathy in HIV-infected patients

**DOI:** 10.1186/s12879-022-07640-8

**Published:** 2022-07-27

**Authors:** Donghe Chen, Yunqi Zhu, Yunbo Chen, Danhua Zhu, Zhengfeng Liu, Tiancheng Li, Yinuo Liu, Kui Zhao, Xinhui Su, Lanjuan Li

**Affiliations:** 1 Department of Nuclear Medicine, The First Affiliated Hospital, Zhejiang University School of Medicine, Hangzhou, 310003 China; 2 State Key Laboratory for Diagnosis and Treatment of Infectious Diseases, The First Affiliated Hospital, Zhejiang University School of Medicine, Hangzhou, 310003 China

**Keywords:** ^18^F-FDG, PET/CT, Diagnosis, Lymphoma, Lymphadenopathy, HIV

## Abstract

**Background:**

It is vital to distinguish between inflammatory and malignant lymphadenopathy in human immunodeficiency virus (HIV) infected individuals. The purpose of our study was to differentiate the variations in the clinical characteristics of HIV patients, and apply ^18^F-FDG PET/CT parameters for distinguishing of malignant lymphoma and inflammatory lymphadenopathy in such patients.

**Methods:**

This retrospective cross-sectional study included 59 consecutive HIV-infected patients who underwent whole-body ^18^F-FDG PET/CT. Of these patients, 37 had biopsy-proven HIV-associated lymphoma, and 22 with HIV-associated inflammatory lymphadenopathy were used as controls. The determined parameters were the maximum of standard uptake value (SUV_max_), SUV_max_ of only lymph nodes (SUV_LN_), the most FDG-avid lesion-to-liver SUV_max_ ratio (SUR_max_), laboratory examinations and demographics. The optimal cut-off of ^18^F-FDG PET/CT value was analyzed by receiver operating characteristic curve (ROC).

**Results:**

Considering the clinical records, the Karnofsky Performance Status (KPS) scores in patients with inflammatory lymphadenopathy were obviously higher than those in patients with malignant lymphoma (P = 0.015), whereas lymphocyte counts and lactate dehydrogenase (LDH) were obviously lower (P = 0.014 and 0.010, respectively). For the ^18^F-FDG PET/CT imaging, extra-lymphatic lesions, especially digestive tract and Waldeyer’s ring, occurred more frequently in malignant lymphoma than inflammatory lymphadenopathy. Furthermore, the SUR_max_ and SUV_LN_ in malignant lymphoma were markedly higher than those in inflammatory lymphadenopathy (P = 0.000 and 0.000, respectively). The cut-off point of 3.1 for SUR_max_ had higher specificity (91.9%) and relatively reasonable sensitivity (68.2%) and the cut-off point of 8.0 for the SUV_LN_ had high specificity (89.2%) and relatively reasonable sensitivity (63.6%).

**Conclusion:**

Our study identified the distinctive characteristics of the clinical manifestations, the SUR_max_, SUV_LN_ and detectability of extra-lymphatic lesions on ^18^F-FDG PET, and thus provides a new basis for distinguishing of malignant lymphoma from inflammatory lymphadenopathy in HIV-infected patients.

## Introduction

HIV is still a major public health problem. There are 37.7 million people living with HIV, including 10.2 million who are not receiving treatment, and there were 1.5 million new HIV infections and 680,000 AIDS-related deaths worldwide in 2020 [1]. Lymphadenopathy is a common manifestation among other consequences of HIV infection. During the highly active anti-retroviral therapy (HAART) era, the main causes of lymphadenopathy were malignant lymphoma and a variety of opportunistic infections (tuberculosis, atypical mycobacteriosis and esophageal candidiasis, etc.) [2, 3]. To this end, it is vital to differentiate malignant lymphoma from inflammatory lymphadenopathy because of different respective treatments and adverse events from delayed diagnosis and overtreatment [4–6].

Conventional clinical imaging modalities are important tools for diagnosing lymphadenopathies. Several imaging methods, including ultrasound, computed tomography (CT) and magnetic resonance imaging (MRI), can be used to determine the condition either as inflammatory or related to tumor. Ultrasound is valuable for the detection of peripheral lymphadenopathies, whereas CT and MRI are employed in evaluating cavitary lymphadenopathies. However, it is still challenging to distinguish benign lymph nodes from malignant ones using the size, location and enhancement mode [7, 8].

As a single and noninvasive examination type, integrated ^18^F-FDG PET/CT has emerged as an important tool for the diagnosis, staging, restaging and treatment monitoring of malignancies by providing functional and morphological information. This technology is also widely used in the management of HIV-infected patients with lymphoma and suspected infection. ^18^F-FDG PET/CT can identify primary cerebral lymphoma from toxoplasmosis-associated lymphoma in AIDS patients, which either CT or MRI cannot reliably achieve [9]. An early study by Mhlanga [10] indicated that quantitative PET metabolic parameters such as metabolic tumor volume (MTV), total lesion glycolysis (TLG) are valuable tools for differentiating lymphoma from reactive adenopathy in HIV-infected patients. However, the measurement of MTV and TLG is still relatively cumbersome and generally suitable for tumors rather than inflammatory lesions.

In this retrospective study, we aimed to characterize the differences in clinical features and ^18^F-FDG PET/CT parameters for distinguishing of malignant lymphoma and inflammatory lymphadenopathy in HIV-infected patients.

## Materials and methods

### Patients

This retrospective, single-institution study was approved by the Clinical Research Ethics Committee of the First Affiliated Hospital, Zhejiang University School of Medicine (No. IIT20220121A). The requirement for informed consent was waived by the Clinical Research Ethics Committee of the First Affiliated Hospital, Zhejiang University School of Medicine because of the retrospective nature of the research.

Fifty-nine HIV-infected patients who had enlarged lymph nodes (diameter ≥ 1 cm) on CT or ultrasound imaging, presenting with fever, fatigue, cough, weight loss, nausea and vomiting, night sweats or local pain, were enrolled in this study from February 2014 and October 2019. Whole-body ^18^F-FDG PET/CT was performed for evaluating the distribution of lymph nodes and selecting the optimal biopsy site.

We identified 37 patients ranging in age from 26 to 75 years (median age, 59 years) with biopsy-proven HIV-associated lymphoma (35 B-cell lymphoma; 1 Hodgkin lymphoma; 1T-cell lymphoma). For 22 HIV-infected patients with biopsy-proven inflammatory lymphadenopathy, 11 patients were infected by *tuberculosis* (n = 5), *nontuberculoaus mycobacteria* (n = 4), *Talaromyces marneffei* (n = 1) and *cryptococcosis* (n = 1) by culture or metagenomics next-generation sequencing (mNGS), but remaining 11 patients were only reactive hyperplasias not found other pathogens infected. After antibiotic therapy or anti-inflammatory treatment, all of these patients had improved in clinical and reimaging with general CT scans. And relapses and new radiological lesions did occur during 3 months follow-up. These patients, ranging in age from 23 to 74 years (median age, 42.5 years), were included in this study as controls.

### PET/CT imaging

All patients were scanned on a unique PET/CT scanner (Biograph 16; Siemens, Germany) in our center. The patients had been fasting for at least 4–6 h, and blood glucose levels were required to be less than 10 mmol/L before ^18^F-FDG injection (3.75–5.55 MBq/kg). Scanning was started from the basal skull and performed to the mid-thigh after an uptake time of 40–60 min. CT scans without intravenous or oral contrast were conducted using a sixteen-slice helical CT with a continuous spiral technique (120 keV; automatic current regulation adjusted to the thickness and density of each patient's body; section thickness of 5 mm). PET scans were obtained for 3 min per frame, and they were reconstructed using an iterative algorithm (Siemens). Additional scans of the extremities were acquired if the patient had tumors or suspected metastasis on the lower extremities below the mid-thigh.

### Measurement of clinical data and metabolic PET parameters

We recorded the age, sex, presenting syndrome, Eastern Cooperative Oncology Group (ECOG), Karnofsky Performance Status score (KPS), duration of HIV infection, duration of anti-HIV therapy, HIV clinical stage of World Health Organization (WHO) on admission, laboratory examination, number of lymph node regions, maximum diameter of lymph nodes, morphology of lymph nodes, FDG accumulation in extra-lymphatic organs, and SUV_max_ value. The laboratory examinations included red cell count, hematocrit, hemoglobin, white blood cell count, neutrophilic granulocyte count, lymphocyte count, platelet count, C-reactive protein (CRP), erythrocyte sedimentation rate (ESR), serum ferritin, LDH, counts of CD4, ratio of CD4 and CD8 count (CD4/CD8), *Mycobacterium tuberculosis* antigen-specific IFN-gamma release assays (T-SPOT) and Epstein-Barr virus detection.

A mannequin based on that by Dana-Farber Cancer Institute was used for counting the number of lymph node regions. Maximum diameter and morphological features (fusion, necrosis and calcification) of lymph nodes were also recorded.

The ^18^F-FDG PET/CT parameters were evaluated visually and semi-quantitatively by the calculation of SUV_max_. SUV_LN_ was defined as the SUV_max_ of only lymph nodes. When multiple sites of abnormal uptake were found in other organs or tissues, SUV_max_ of the most FDG-avid lesion was used for subsequent analysis. In addition, the most FDG-avid lesion-to-liver SUV_max_ ratio was defined as SUR_max_. Other parameters included SUV_Liver_ (SUV_max_ of liver), SUV_Spleen_ (SUV_max_ of spleen) and SUV_Marrow_ (SUV_max_ of marrow). Experienced nuclear medicine physicians used Medex Electric Medical Recording System (MEMRS PACS), which provides manual delineation of the region of interest (ROI) and automatic generation of SUV_max_ of ROI, in order to evaluate the initial investigation of ^18^F-FDG PET/CT images.

### Statistical analysis

All statistical analyses were carried out using SPSS Statistics software (version 20; SPSS Inc. Chicago, USA). The analysis of differences was performed with chi-square or Fisher’s exact test. A P-value of less than 0.05 was considered statistically significant. ROC curves were used to determine the optimal cut-off values.

## Results

### Demographics and clinical features

The clinical features of HIV-infected patients with malignant lymphoma and those with inflammatory lymphadenopathy are summarized in Table 1. The demographic data, clinical features and laboratory examinations of the two groups did not differ significantly with regards to the presenting syndrome, duration of HIV infection, duration of anti-HIV therapy, HIV stages on admission, white cell counts, neutrophil counts, platelet counts, red-cell counts, hemoglobin, CRP, ESR, Ferritin, CD4 count, ratio of CD4/CD8 and detection of EBV. Patients with malignant lymphoma had lower ECOG and KPS score than those with inflammatory lymphadenopathy (P = 0.013 and P = 0.015). The lymphocyte counts and LDH in patients with malignant lymphoma were higher than those in patients with inflammatory lymphadenopathy (P = 0.014 and 0.010, respectively). The median lymphocyte counts of malignancy lymphoma group and inflammatory lymphadenopathy were 1.1 (range, 0.1–2.4) and 0.7 (range, 0.2–1.8), respectively. The lymphocyte counts were still at low levels (< L1.0 × 10^9^/L) in most patients (19/37 patients with malignancy lymphoma and 16/22 patients with inflammatory lymphadenopathy). Eight of 22 patients (36.4%) with inflammatory lymphadenopathy were positive for T-SPOT testing, which was only three in 37 (8.1%) among malignant lymphoma patients.

**Table 1:** Comparison clinical and FDG PET/CT characteristics between HIV-infected patients with malignant lymphoma and those with inflammatory lymphadenopathy

Characteristics	Malignant lymphoma (n = 37)	Inflammatory lymphadenopathy (n = 22)	P
Sex, n (%)			
Male/female	33 (89.2%)/4 (10.8%)	19 (86.4%)/3 (13.6%)	0.751
Median age (y)	43 (26–75)	42.5 (23–74)	0.714
Presenting syndrome, n (%)			
Fever (> 38℃)	12 (32.4%)	12 (54.5%)	0.109
Fatigue	6 (16.2%)	8 (36.4%)	0.114
Cough	5 (13.5%)	9 (40.9%)	0.055
Weight loss	14 (37.8%)	10 (45.5%)	0.789
Nausea and vomiting	3 (8.1%)	2 (9.1%)	1.000
Night sweats	3 (8.1%)	2 (9.1%)	1.000
Local pain	15 (40.5%)	6 (27.3%)	0.402
KPS, median (interquartile range)	80 (40–90)	90 (70–90)	0.015*
ECOG, n (%)			0.013*
0/1	23 (62.2%)	19 (86.4%)	0.074
2/3	14 (37.8%)	3 (13.6%)	0.074
4/5	0 (0%)	0 (0%)	NA
Duration of HIV infection (months), median (interquartile range)	3 (0–96)	7 (0–108)	0.303
Duration of anti-HIV therapy (months), median (interquartile range)	2(0–96)	6 (0–108)	0.193
WHO clinical stage of HIV on admission, n (%)			0.844
I	16 (43.2%)	8 (36.4%)	0.785
II	3 (8.1%)	3 (13.6%)	0.666
III	9 (24.3%)	6 (13.6%)	1.000
IV	9(24.3%)	5 (22.7%)	0.748
Laboratory examination, median (interquartile range)			
White cell count (10E9/L)	4.6 (0.5–35.9)	3.5 (1.9–8.6)	0.218
N (10E9/L)	2.4 (0.30–7.7)	1.8 (1.1–6.9)	0.707
L (10E9/L)	1.1 (0.1–2.4)	0.7 (0.2–1.8)	0.014*
Platelet count (10E9/L)	148.0 (14.0–429.0)	3.2 (2.2–5.0)	0.563
Red-cell count(10E9/L)	3.70 (2.06–5.43)	198.0 (36.0–414.0)	0.121
Hematocrit (%)	33.9 (18.5–44.9)	31.3 (21.9–44.5)	0.462
Hemoglobin (g/dL)	116.0 (63.0–158.0)	110.1 (65.0–152.0)	0.533
C-reactive protein (mg/L)	18.3 (0–218.8)	28.2 (0.6–125.2)	0.304
Erythrocyte sedimentation rate (mm/H)	21.0 (2.0–105.0)	29.0 (3.0–117.0)	0.779
Lactate dehydrogenase (U/L)	465 (134–8396)	218 (139–876)	0.010*
Ferritin (ng/mL)	398.8 (7.4–40,000.0)	534.9 (66.8–3524.0)	0.385
CD4 (cells/μL)	128 (4–714)	137 (10–399)	0.910
CD4/CD8	0.25 (0.01–0.87)	0.28 (0.02–1.47)	0.255
T-spot (+), n (%)	3 (8.1%)	8 (36.4%)	0.013*
EBV (+), n (%)	22 (59.4%)	9 (40.9%)	0.189
Numbers of lymph node involved areas median (interquartile range)	6 (1–11)	3 (1–9)	0.006*
Maximum diameter of lymph nodes (cm), median (interquartile range)	4.0 (1.0–19.1)	1.8 (1.1–5.8)	0.001*
Morphological features of lymph nodes, n%			
Fusion	17 (45.9%)	3 (13.6%)	0.021
Necrosis	4 (10.8%)	6 (27.3%)	0.152
Calcification	0 (0%)	2 (9.1%)	0.135
FDG accumulation in extra-lymphatic organs, n%	31 (83.8%)	12 (54.5%)	0.000*
Bone marrow	16 (43.2%)	4 (18.2%)	0.098
Spleen	10 (27.0%)	9 (9.1%)	0.388
Digestive tract	11 (29.7%)	0 (0%)	0.004*
Waldeyer’s ring	13 (35.1%)	2 (9.1%)	0.033*
Liver	11 (29.7%)	1 (4.5%)	0.053
Nasal and sinuses	3 (8.1%)	0 (0%)	1.000
Pancreas	3 (8.1%)	1 (4.5%)	1.000
Adrenal	3 (8.1%)	0 (0%)	0.286
Skin	2 (5.4%)	0 (0%)	0.524
Peritoneum	2 (5.4%)	0 (0%)	0.524
CNS	1 (2.7%)	0 (0%)	1.000
SUV measurement, median (interquartile range)			
SUV_LN_	18.5 (3.6–32.0)	5.2 (1.3–22.6)	0.000*
SUV_Marrow_	3.1 (1.3–21.8)	3.2 (1.6–8.2)	0.002*
SUV_Spleen_	2.4 (1.3–12.2)	2.4 (1.6–7.9)	0.700
SUV_Liver_	2.7 (1.3–16.3)	2.6 (1.7–4.7)	0.017*
SUR_max_	7.9 (2.1–15.5)	3.0 (0.6–8.4)	0.000*

HIV: human immunodeficiency virus; WHO: World Health Organization; KPS: Karnofsky Performance Status score; ECOG: Eastern Cooperative Oncology Group; FDG: ^18^F-2-fluoro-2-deoxy-d-glucose; CNS: central nervous system; SUV: standard uptake value; SUV_LN_: the maximum of standard uptake value of only lymph nodes; SUR_max_: the most FDG-avid lesion-to-liver SUV_max_ ratio. SUV_Liver_: SUV_max_ of liver; SUV_Spleen_: SUV_max_ of spleen; SUV_Marrow_: SUV_max_ of bone marrow

^*^P value less than 0.05 was considered statistically significant

### ^18^F-FDG PET/CT findings in HIV-infected patients with malignant lymphoma

**Fig. 1:**
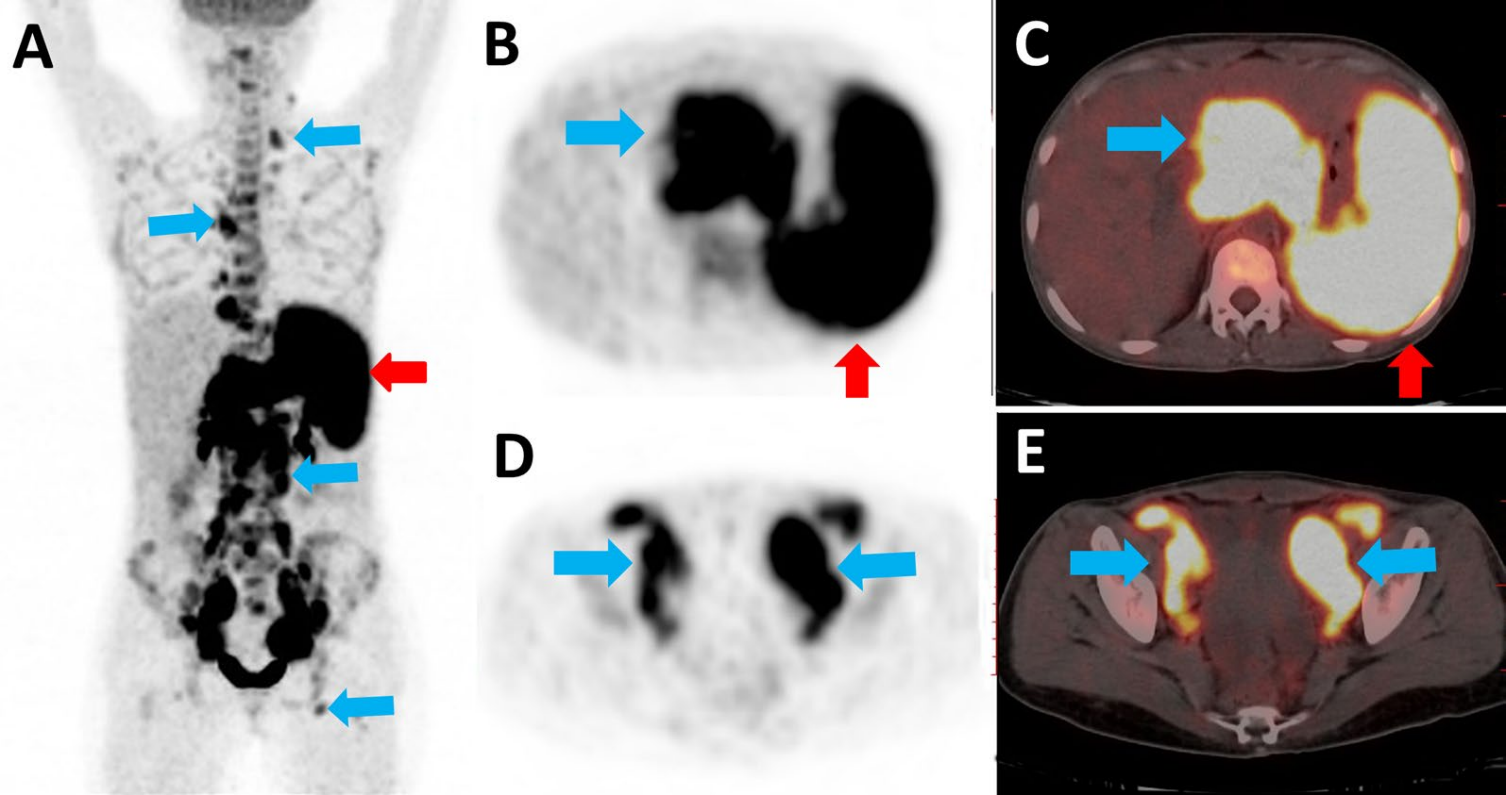
A 27-year-old female patient with a two-year history of HARRT presented with upper abdominal pain, vomiting and diarrhea. The initial ^18^F-FDG PET/CT maximum intensity projection (**A** PET) and axial slices (**B**, **D** PET; **C**, **E** PET/CT) showed hyper-metabolic lymph nodes in the neck, mediastinum and retro-peritoneum (blue arrows) in addition to the spleen (red arrows). The SUV_max_ of lymph nodes and spleen was 18.6 and 8.8, respectively. The neck lymph node biopsy confirmed diffuse large B-cell lymphoma

A total of 31 out of 37 patients were found to have multiple lymph nodes areas involved with median numbers of 6 areas (range, 1–11) (Fig. 1A–E, blue arrows). The median maximum diameter of lymph nodes in these patients was 4.0 cm (range, 1.0–19.1). The manifestations of lymph node fusion were common in 17 out of 37 patients and necrosis and calcification were rare (4/37, 0/37).

Abnormal FDG accumulation was found in 31/37 patients (91.3%) with extra-lymphatic lesions. Bone marrow was the most commonly involved organ in 16/37 patients (43.2%), followed by Waldeyer’s ring (n = 13, 35.1%), digestive tract (n = 11, 29.7%), liver (n = 11, 29.7%) and spleen (n = 10, 27.0%) (Fig. 1A–C, red arrows). Rare extra-lymphatically involved organs included those in the nasal region and sinuses (n = 3), adrenal gland (n = 3), pancreas (n = 3), skin (n = 2), peritoneum (n = 2) and central nervous system (n = 1).

In all 37 patients with malignant lymphoma, ^18^F-FDG PET/CT revealed intense or moderate FDG accumulation in the lymph nodes, and the median SUV_max_ was 18.5 (range, 3.6–32.0). The median SUV_max_ of the bone marrow, liver and spleen in these patients were 3.1 (range, 1.3–21.8), 2.7 (range, 1.3–16.3) and 2.4 (range, 1.3–12.2), respectively. The median SUR_max_ value was 7.9 (range, 2.1–15.5).

### ^18^F-FDG PET/CT findings for HIV-infected patients with inflammatory lymphadenopathy

**Fig. 2:**
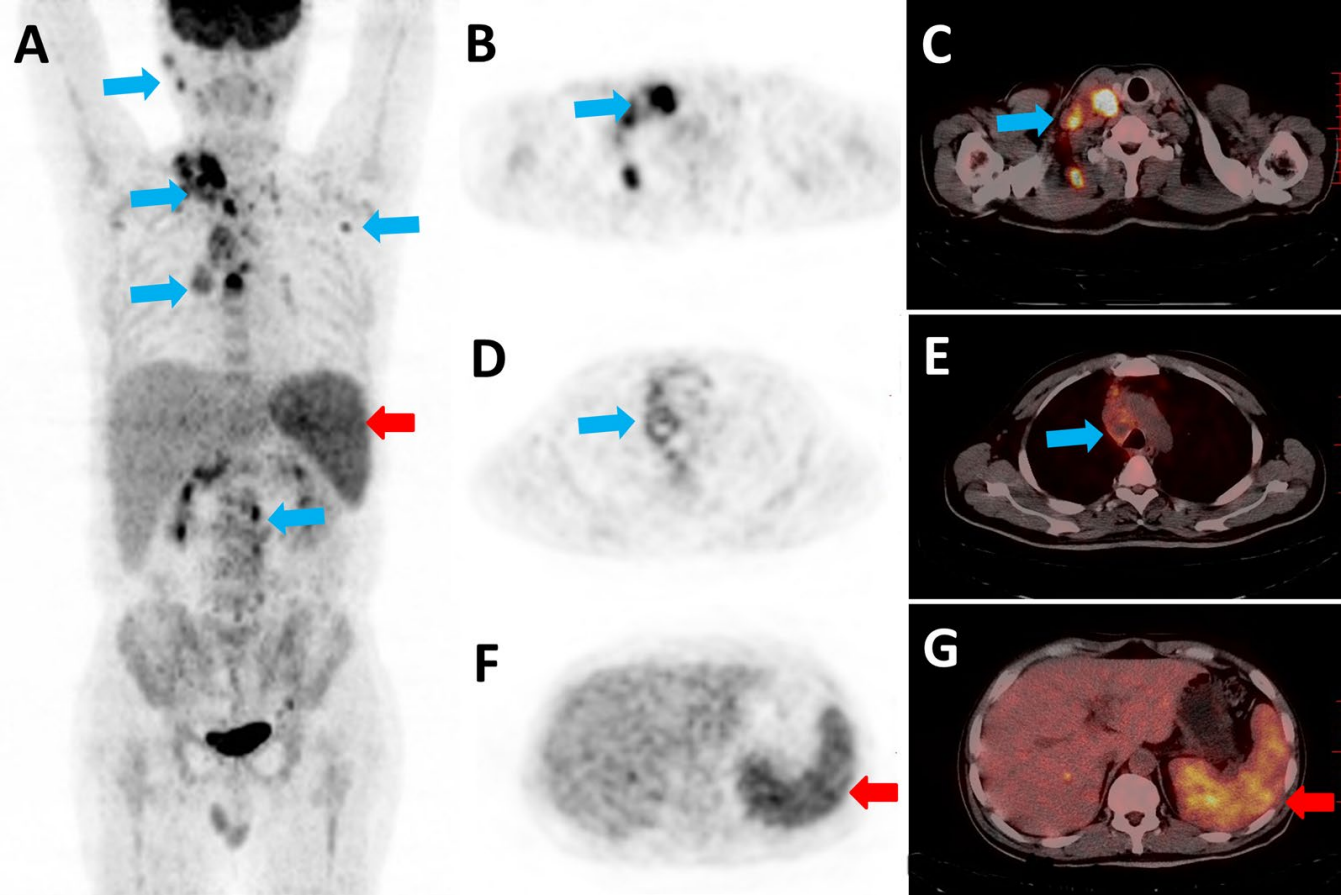
A 47-year-old male patient with no previous relevant history, presenting with cervical painful swollen lymph nodes with intermittent fever for 3 months. Finally, HIV infection was confirmed at the local Centers for Disease Control (CDC). Blood test showed Epstein-Barr virus infection by polymerase chain reaction, and the T-SPOT were positive. An ^18^F-FDG PET/CT was performed, with maximum intensity projection (**A**) and axial slices (**B**, **D** and **F** PET; **C**, **E** and **G** PET/CT), showing cervical, mediastinal and retroperitoneal lymph nodes high uptake (blue arrows) and splenomegaly moderate uptake (red arrows). The axial slices (**B** and **C**, blue arrows) show cervical node involvement, with SUV_max_ value of 9.2. Multiple necroses were found in the mediastinal lymph nodes (**D** and **E**; blue arrows). Right neck lymph node biopsy confirmed granulomatous lymphadenitis with positive bacteria by Ziehl–Neelsen staining. And the specimen culture proved to be *Mycobacterium tuberculosis* infection finally

Multiple lymph node areas were involved in 14/22 patients with median numbers of 3 areas (range, 1–9) (Fig. 2A–E). The median maximum diameter of lymph nodes in these patients was 1.8 cm (range, 1.1–5.8). The manifestations of lymph nodes necrosis (Fig. 2D, E) were more common in patients with inflammatory lymphadenopathy than those with malignant lymphoma (27.3% vs. 10.8%). The manifestations of lymph node fusion and calcification were not common (3/22, 2/22).

The ^18^F-FDG PET/CT indicated abnormal FDG uptake by extra-lymphatic lesions in 12/22 patients (54.5%). The spleen was the most commonly involved organs in 9/22 patients (40.9%) (Fig. 2A, F, G, red arrows), followed by bone marrow (n = 4) and Waldeyer’s ring (n = 2). Rare extra-lymphatic involved organs included the liver (n = 1) and pancreas (n = 1). No abnormal FDG uptake was observed in the adrenal gland, skin, peritoneum and CNS.

The ^18^F-FDG PET/CT revealed moderate or high FDG uptake by the lymph nodes in most patients (20/22, 90.0%), and the median SUV_max_ was 5.2 (range, 1.3–22.6). The median SUV_max_ of the bone marrow, liver and spleen in these patients were 3.2 (range, 1.6–8.2), 2.6 (range, 1.7–4.7) and 2.4 (range, 1.6–7.9), respectively. The median SUR_max_ was 3.0 (range, 0.6–8.4).

### Comparing of FDG uptake between HIV-infected patients with malignant lymphoma and those with inflammatory lymphadenopathy

The characteristics and differences of PET/CT parameters in HIV-infected patients with malignant lymphoma and those with inflammatory lymphadenopathy were showed in Table 1. The number of lymph node-involved areas and maximum diameter of lymph nodes were significantly different between patients with malignant lymphoma and those with inflammatory lymphadenopathy, with P values of 0.006 and 0.001, respectively. Lymph node fusion was seen more frequently in patients with malignant lymphoma than in those with inflammatory lymphadenopathy (45.9% vs. 13.6%) with statistical significance (P = 0.021). Malignant lymphoma invaded more commonly to extra-lymphatic organs than inflammatory lymphadenopathy with obvious statistical significance (83.8% vs. 54.5%, P = 0.000). In addition, the involved organs of the digestive tract and Waldeyer’s ring differed significantly between the HIV-infected two groups, with P values of 0.004 and 0.033, respectively.

**Fig. 3:**
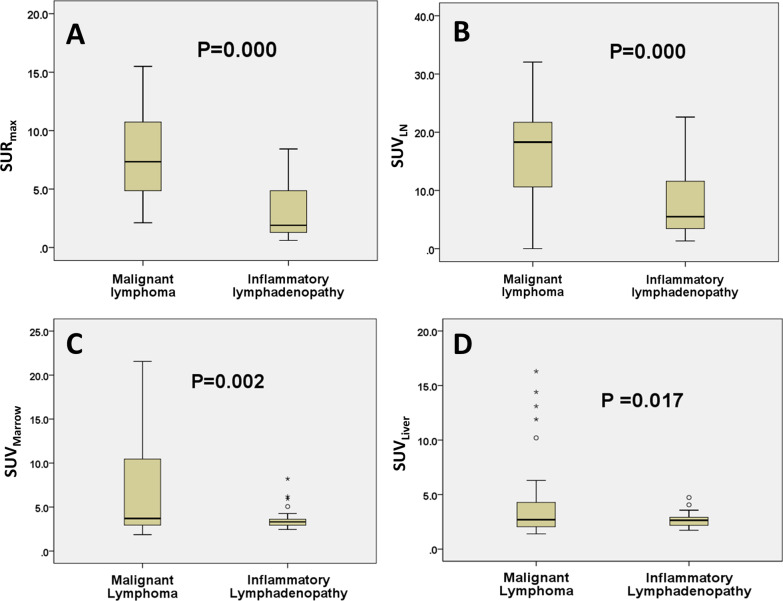
Association between lymphoma status and PET parameters established by using a gradient-based segmentation method. The correlations are shown for SUR_max_ (**A**), SUV_LN_ (**B**), SUV_Marrow_ (**C**) and SUV_Liver_ (**D**)

The SUR_max_ value in patients with malignant lymphoma were higher than that in patients with inflammatory lymphadenopathy (P = 0.000) (Fig. 3A). The SUV_LN_, SUV_Marrow_ and SUV_Liver_ in patients with malignant lymphoma were higher than that in patients with inflammatory lymphadenopathy, with P values of 0.000, 0.002 and 0.017, respectively (Fig. 3B–D).

**Fig. 4:**
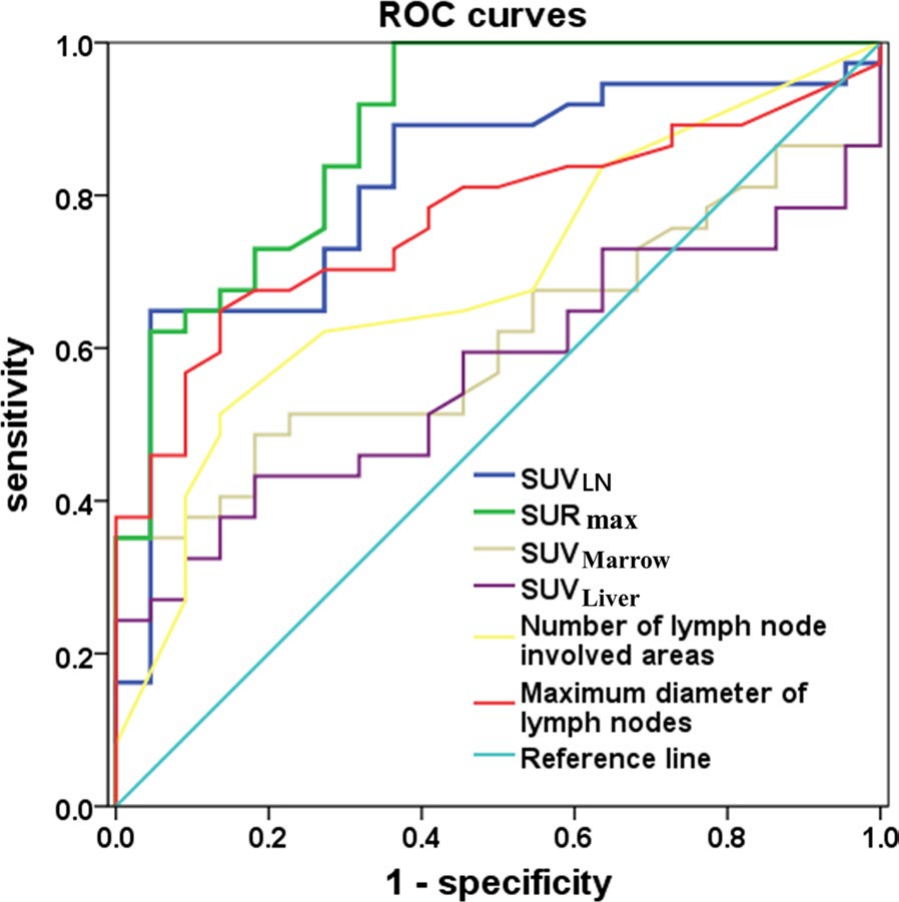
ROC curve for PET parameters as a screening test for malignant lymphoma and inflammatory lymphadenopathy. The discriminatory ability of SUR_max_ and SUV_LN_ was better than that of the maximum diameter and involved areas of lymph nodes, the SUV_Marrow_ and SUV_Liver_ in malignant lymphoma and inflammatory lymphadenopathy

The ROC curves and cut-off value of different parameters in HIV-infected patients with malignant lymphoma and those with inflammatory lymphadenopathy were analyzed (Table 2). The areas under the ROC curves were 0.888 for SUR_max_ (P < 0.001) and 0.815 for SUV_LN_ (P < 0.001), but 0.611 for SUV_Marrow_ (P = 0.156) and 0.567 for SUV_Liver_ (P = 0.393) (Fig. 4). The cut-offs showing the best equilibrium between sensitivity and specificity approached 3.1 and 8.0 mmol/L for SUR_max_ and SUV_LN_ respectively. The cut-off point of 8.0 had also high specificity (89.2%) for the SUV_LN_ with relatively reasonable sensitivity (63.6%). Furthermore, the cut-off point of 4.0 had higher specificity (91.9%) and sensitivity (68.2%) for SUR_max_ than those of simply using the SUV_LN_. The areas under the ROC curves were 0.888 for numbers of involved areas and 0.815 for maximum diameters of lymph node (P < 0.05). The cut-offs with the best equilibrium between specificity and sensitivity approached 5 for numbers of involved areas and 3.6 cm for maximum diameters of lymph node, but with relatively low specificity (72.7% and 86.4%) and sensitivity (62.2% and 64.9%).

**Table 2:** The ROC curves and cut-off value of different parameters in HIV-infected patients with malignant lymphoma and those with inflammatory lymphadenopathy

parameter	Areas under the ROC curve	P value	Data of cut-off value		
			Cut-off value	Sensitivity	Specificity
SUR_max_	0.888	0.000	3.1	68.2%	91.9%
SUV_LN_	0.815	0.000	8.0	63.6%	89.2%
SUV_Marrow_	0.611	0.156	NA*	NA	NA
SUV_Liver_	0.567	0.393	NA	NA	NA
Number of lymph node involved areas	0.692	0.014	5	62.2%	72.7%
Maximum diameter of lymph nodes	0.768	0.001	3.6	64.9%	86.4%

ROC: receiver operating characteristic curve; HIV: human immunodeficiency virus; SUV: standard uptake value; SUVLN: the maximum of standard uptake value of only lymph nodes; SUR_max_: the most FDG-avid lesion-to-liver SUV_max_ ratio. SUV_Liver_: SUV_max_ of liver; SUV_Spleen_: SUV_max_ of spleen; SUV_Marrow_: SUV_max_ of bone marrow

NA*: no statistical significance and cut-off value were found

## Discussion

Lymphadenopathy is the most characteristic clinical manifestation in HIV-infected patients, as lymphoid tissue is a major target of HIV [10]. The early and accurate diagnosis of lymphadenopathy is essential to formulation of an efficient treatment plan. However, the distinguishing of benign and malignant enlarged lymph nodes has always been difficult, no matter whether in patients with HIV-infected or not. Clinicians should comprehensively evaluate the results of imaging and laboratory examination, and make rapid decisions on the biopsy of suspicious lymph nodes or extra-lymphatic lesions to achieve timely diagnosis and treatment.

Clinical characteristics and laboratory examination results including treatment history, HIV stage, duration of HIV infection and LDH are important predictors of disease outcome in HIV-infected patients with lymphoma [4, 11]. In our study, patients with malignant lymphoma had lower KPS scores and higher LDH level than those with inflammatory lymphadenopathy. Low KPS score and high LDH level in HIV-patients have suggested severe disease or high tumor burden by malignant lymphoma, and consequently the need for the attention of clinicians. For the analysis of lymphocyte counts, we also found that lymphocyte counts were higher in the malignancy malignant group than inflammatory lymphadenopathy group with statistically significance. We considered that high invasive lymphoma involvement of the bone marrow or spleen may cause elevated peripheral blood lymphocytes including heterogeneous lymphocytes. However, lymphocyte counts were still at low levels in most patients (19/37 patients with malignancy lymphoma and 16/22 patients with inflammatory lymphadenopathy). Therefore, its value in distinguishing of these two diseases and relationship with HIV viral loads still needed to further study.

The laboratory examination of a patient’s immune status such as CD4 count and CD4/CD8 can indicate the disease activity and offer information of clinical significance [12]. CD4 counts have been inversely correlated with the uptake of FDG by the lymph nodes [13–15]. Furthermore, some studies [4, 16] considered that the degree of immunosuppression with median lymphocyte CD4 count (< 200 cells/mm^3^) is proportional to the risk of developing lymphoma. However, in this study, CD4 count and CD4/CD8 did not help to distinguish malignant lymphoma and inflammatory lymphadenopathy, which is in concordance with the previous report by Mhlanga [10]. Prospective, large samples and multi-center study was needed to further explore the relationship between CD4 count and malignant lymphoma in HIV-infected patients.

Whole-body ^18^F-FDG-PET/CT scan detected more involved lymph nodes than localized CT and ultrasound [17]. Lesions with intense uptake of FDG or visual greater than liver had a positive predictive value of 95% for pathology, indicating the need for treatment [17, 18]. In our research, the number of lymph node involved areas and the maximum diameter of lymph nodes assessed by ^18^F-FDG PET/CT in malignant lymphoma patients were significantly higher and larger, respectively, than that in inflammatory lymphadenopathy patients. Using a cut-off value of 5 for the number of lymph node involved areas and 3.6 for maximum diameter of lymph nodes allowed for separation of those two groups, but with inferior sensitivity and specificity.

Our findings revealed that FDG accumulation in extra-lymphatic organs was more frequent in malignant lymphoma patients, especially in the digestive tract and Waldeyer’s ring, strongly suggesting the possibility of malignant lymphoma. However, FDG accumulation in the spleen and bone marrow were common in both malignant lymphoma and inflammatory lymphadenopathy, and there was no clinical significance in distinguishing those diseases, contrary with the previous literature [8].

The semi-quantitative analysis of standard uptake value (SUV) can improve diagnostic specificity, but there is no certain cut-off value [19, 20]. In our study, the cut-off point of 8.0 for the SUV_LN_ had high specificity (89.2%) but relatively low sensitivity (63.6%). Indeed, there are some limitations in using SUV_max_ of the lymph nodes alone, because the most FDG-avid of involved lesions often occurred in extra-lymphatic organs.

Liver standard uptake is often used as reference criteria for radioactive distribution due to stable FDG uptake. Studies showed that the standard uptake ratio (SUR) is superior to SUV as a replaceable parameter of the FDG metabolic rate [21, 22]. Therefore, we defined a new parameter SUR_max_ by calculating the ratio of the SUV_max_ of involved lesions (lymph nodes or extra-lymphatic organs) and the liver standard uptake to improve diagnostic efficiency. The cut-off point of 3.1 for SUR_max_ had higher specificity (91.9%) and sensitivity (68.2%) than those of simply using the SUV_LN_. The effect was close to that of contrast-enhanced endoscopic ultrasound fine-needle in lymph nodes with a sensitivity of 85.9–94.3% and a specificity of 75.1–87.7% [23]. Therefore, the cut-off point of 3.1 for SUR_max_ can be a new basis for distinguishing of malignant lymphoma from inflammatory lymphadenopathy in HIV-infected patients.

We recognize the following limitations of our study: first, it was performed at a single center and the sample size was relatively small; multicenter analysis will be designed in a future study to achieve a better representation of lymphadenopathy in HIV-infected patients. Second, this was a retrospective study, and the use of ^18^F-FDG PET/CT in the assessment of patients produced a potential bias toward an advanced malignant lymphoma cohort. Third, patients with lymphadenopathy by other HIV-associated malignancies, such as Kaposi’s sarcoma or cancer, were not enrolled. Therefore, our findings may not be suitable for distinguishing of malignant lymphoma and Kaposi's sarcoma or cancer.

## Conclusions

This study identified the distinctive features of clinical characteristics and the SUR_max_, SUV_LN_ of ^18^F-FDG-PET, which provides a new basis for distinguishing of malignant lymphoma from inflammatory lymphadenopathy in HIV-infected patients. Using the cut-off point of 3.1 for SUR_max_ or 8.0 for SUV_LN_ can be a sensitive and easy method to discriminate between malignant lymphoma and inflammatory lymphadenopathy. Moreover, FDG accumulation in the digestive tract and Waldeyer’s ring, KPS score, lymphocyte counts and LDH also has a significant importance in distinguishing these diseases. On the other hand, the patient’s immune status, SUV_max_ of the bone marrow and spleen, which are commonly used by nuclear medicine and clinicians, are not helpful in distinguishing of these diseases.

## Data Availability

All data generated or analyzed during this study are included in this published article.
